# Preliminary Study on Nutritional Value and Biologically Active Components of Kidney Vetch (*Anthyllis vulneraria* L.)

**DOI:** 10.3390/plants15131954

**Published:** 2026-06-25

**Authors:** Olga Teneva, Zhana Petkova, Ginka Antova, Maria Angelova-Romova, Elis Yusein, Tsvetelina Mladenova, Donika Gyuzeleva, Anelia Bivolarska, Rumen Mladenov, Krasimir Todorov, Plamen Stoyanov

**Affiliations:** 1 Department of Chemical Technology, Faculty of Chemistry, University of Plovdiv “Paisii Hilendarski”, 24 Tzar Asen Str., 4000 Plovdiv, Bulgaria; zhanapetkova@uni-plovdiv.bg (Z.P.); ginant@uni-plovdiv.bg (G.A.); maioan@uni-plovdiv.bg (M.A.-R.); stu2204221006@uni-plovdiv.bg (E.Y.); 2 Department of Botany and Biological Education, Faculty of Biology, University of Plovdiv “Paisii Hilendarski”, 24 Tzar Asen Str., 4000 Plovdiv, Bulgaria; cmladenova@uni-plovdiv.bg (T.M.); dgyuzeleva@uni-plovdiv.bg (D.G.); rummlad@uni-plovdiv.bg (R.M.); ktodorov@uni-plovdiv.bg (K.T.); pstoyanov@uni-plovdiv.bg (P.S.); 3 Department of Bioorganic Chemistry, Faculty of Pharmacy, Medical University of Plovdiv, Vasil Aprilov Str. 15A, 4002 Plovdiv, Bulgaria; 4 Department of Medical Biochemistry, Faculty of Pharmacy, Medical University of Plovdiv, Vasil Aprilov Str. 15A, 4002 Plovdiv, Bulgaria; anelia.bivolarska@mu-plovdiv.bg

**Keywords:** *Anthyllis vulneraria*, nutrients, glyceride oil, fatty acids, sterols, tocopherols

## Abstract

The aim of the current study is to determine the nutritional value and the content of the biologically active components in kidney vetch (*Anthyllis vulneraria* L.). It is established that the dry biomass contains substantial amounts of proteins and carbohydrates, primarily dietary fiber, while the total oil content is relatively low (below 3.0%). The isolated glyceride oil represents the complete lipid fraction derived from all plant parts (leaves, stems, and flowers). The glyceride oil of *A. vulneraria* is notable for its high levels of biologically active constituents, particularly sterols, tocopherols, and phospholipids. Palmitic (30.3%) and oleic (11.5%) acids dominate the fatty acid profile; *β*-sitosterol, *α*-tocotrienol, and *α*-tocopherol are the major sterol and tocopherol components, respectively. On the other hand, phosphatidylinositol, together with phosphatidic acids, prevails within the phospholipid fraction. Based on the obtained fatty acid composition, several important ratios were calculated—unsaturated fatty acids (UFA)/saturated fatty acids (SFA), saturated fatty acids/monounsaturated fatty acids (MUFA), polyunsaturated fatty acids (PUFA)/saturated fatty acids, and n-6/n-3, providing an integrated assessment of the lipid quality. The PUFA/SFA value (0.24) suggests relatively high oxidative stability. In contrast, the n-6/n-3 ratio (0.86) shows a balanced distribution of essential fatty acids, which is associated with favorable nutritional properties.

## 1. Introduction

The search for new, non-traditional sources of nutritional and medicinal compounds is becoming increasingly important. Although conventional crops supply essential nutrients, their production costs remain high, and there is a growing demand for natural plant-based alternatives to synthetic pharmaceuticals [1]. This underscores the need to identify novel and economically viable similar crops. The species *Anthyllis vulneraria* L. (family Fabaceae) shows promising potential in this regard. This plant is a highly variable (polymorphic) species; therefore, many of its subspecies and varieties have been described. In Bulgaria, it is currently believed to be represented by five subspecies and *Anthyllis vulneraria* ssp. *vitellina* (Velen.) is the most popular [2,3]. It is distributed in Europe, the Mediterranean, Southwest Asia, and the Caucasus.

*A. vulneraria* is not considered an oil-bearing plant, but small amounts of glyceride lipids are still present in all aerial parts, although in different quantities. As in most members of the Fabaceae family, the seeds contain the most concentrated lipid fraction. The lipid content in the leaves and flowers is moderate; these plant organs contain mainly structural lipids—phospholipids, glycolipids, and a small amount of triglycerides. The chemical composition of the aerial organs of *A. vulneraria* varies considerably. The leaves are reported to contain higher levels of phenolic compounds, whereas the flowers are characterized by waxes, small amounts of triacylglycerols, and lipophilic pigments, which have been associated with potential biological activity [4,5]. In contrast, the stems and roots contain predominantly structural lipids and generally exhibit the lowest total lipid content, which is why these organs are less frequently subjected to detailed lipid analysis.

*A. vulneraria* is a well-known medicinal herbaceous plant, growing in grassy places in meadows and pastures, in thickets and thin forests, on rocky slopes and high mountain meadows, from the plains to the high mountain belt, most often on limestone to 3000 m in elevation. The stem is prostrate, erect, and branched, from 5 to 60 cm long. The flowers are collected in heads of inflorescences at the top of the stems, and at the base are covered with two palmately divided bracts. It blooms from May to September [2,3].

The most common use of *A. vulneraria* is for the treatment of skin injuries, using mainly the flowers, followed by the aerial parts and leaves [1]. The research on this plant has been mainly focused on antioxidant and wound-healing properties, which are driven primarily by phenolics and flavonoids. There are some authors who give information about the content of polyphenols and flavonoids in the flowers of *A. vulneraria* [1]. Other researchers have analyzed the aerial parts of the plant and have examined the phytochemical profile of *A. vulneraria* extracts, revealing high concentrations of saponins, tannins, and carotenoids—bioactive constituents widely recognized for their strong antioxidant activity [6,7].

The available literature indicates a lack of data on the lipid composition of the individual organs of *A. vulneraria*, as well as an absence of information regarding the characteristics of its glyceride oil and its biologically active constituents. In addition, there is a continuous search for new plant-derived raw materials that provide higher levels of essential fatty acids and key bioactive compounds, such as tocopherols, phospholipids, and sterols. The essential fatty acids cannot be synthesized by the human body and must be obtained exclusively through the diet, making their balanced intake crucial for maintaining health and preventing various disorders. Addressing these gaps, the present study provides the first detailed characterization of the overall lipid composition of this species, which may support its consideration as a potential non-traditional source from a nutritional and pharmacological point of view. Therefore, the aim of this study was to characterize the proximate chemical composition of *A. vulneraria*, to perform a comprehensive analysis of the lipid composition of its glyceride oil, and to assess its potential relevance for applications in the food and pharmaceutical industries.

## 2. Results and Discussion

The proximate chemical composition of the plant species *A. vulneraria* was investigated. The results are presented in Table 1.

As a typical member of the Fabaceae family, the species shows a low oil content (2.3%) but remains interesting for further analysis of its fatty acid profile, particularly regarding essential fatty acids. According to Lamce et al. [8], the oil content of peas was found to be 2.14%, which is comparable to the value reported in our study, whereas the oil content of lentils is approximately three times lower (0.65%). Other reports declared that total lipid levels in Fabaceae grains typically range from 1.7% to 4.5% [9]. This thesis is in agreement with Boschin and Arnoldi [10], who presented the content of glyceride oil from 3.8% to 4.6% for the sainfoin seeds, lentil (1.30–1.59%), broad bean (1.24–1.75%), and common bean (1.95–2.99%). Some researchers have investigated the oil content of the non-traditional legume crop lupin, reporting 7.4% oil in the seeds [11]. In conclusion, the overall glyceride oil content plays a major role in determining the nutritional value of the seeds and often includes a wide array of lipid-soluble bioactive compounds, such as tocopherols, carotenoids, and sterols. These constituents possess antioxidant activity and can contribute to improving human health.

The analyzed sample is characterized by a relatively high proportion of proteins (13.7%), which contributes to its favorable nutritional profile. Marinas and García-González [12] investigated the plant *A. vulneraria* and determined protein content to be 14.2% (June) and 13.9% (July). Data is close to our results, where the total amount of protein in June was 13.7%. Elamine et al. [13] reported that the protein content of *A. vulneraria* seeds was 23.09%, which is almost twice the value obtained in the present study for the whole aerial parts of the plant. According to several other authors, the total protein content in Fabaceae seeds is as follows: sainfoin seeds contain 25.8–26.8% protein [14], while chickpea, pea, and lentil seeds contain 15.5–28.2%, 18.3–31.0%, and 23.0–32.0%, respectively [15]. Based on these data, the aerial parts of *A. vulneraria* can be described as having relatively high protein levels, within the range reported for the species. This is particularly relevant in the context of promoting sustainable alternatives to animal-derived proteins.

The total carbohydrates in the plants were 67.9% on a fresh basis, while the dried ones possessed slightly higher content (76.0%). Compared to the seeds from other species of the legume family, the examined plant is characterized by a higher carbohydrate content: dry bean (53%) and chickpea (54%) [16]; sainfoin seeds (55.1–57.2%) [14]; mung beans (62.3%) [17]. A significant portion of these carbohydrates consists of crude fiber (31.0%), which is associated with benefits for digestive and metabolic health. Similar to our results, other scientists report neutral detergent fiber content for the same plant as follows: 43.8% (June) and 36.2% (July) [12].

The ash content (5.5%) and moisture level (10.6%) in the *A. vulneraria* fall within the typical ranges for dried plant material. The ash content represents the total amount of inorganic residue remaining after complete combustion of the plant materials and is a primary indicator of the overall content of the minerals present in the samples. The energy value of the species was calculated on the basis of the content of total proteins, total glyceride oil, and the difference between the total carbohydrates and fiber content. It is established that the caloric value is 223.1 kcal/100 g on a fresh basis and 251.1 kcal/100 g on a dry weight. The obtained data indicate that *A. vulneraria* contains a nutritionally balanced proportion of carbohydrates, proteins, and lipids, which defines its overall nutritional profile.

The content of unsaponifiable matter, total sterols, tocopherols, and phospholipids, which are part of the lipid-soluble biologically active components, is presented in Table 2.

The unsaponifiable lipid fraction is a potential source of bioactive components, including phytosterols and tocopherols. These compounds possess well-documented anti-oxidant, anti-inflammatory, and hypocholesterolemic properties [18]. The high levels of sterols and tocopherols indicate that the oil contains compounds typically associated with antioxidant effects. The isolated oil is rich in unsaponifiable matter, which accounts for 28.5% of its composition. This amount is considerably higher than the values reported in Codex-Stan 210 [19] for traditional seed oils (0.9–2.0%), while in the bitter vetch seed oil (family Fabaceae), the unsaponifiable fraction reaches 39.7% [20]. The total sterol content is 7.8%, while according to Schlag and Vetter [21], the phytosterol content of edible oils generally ranges from 0.1 to 1.0%, with legume oils typically falling within the 0.2–0.35% range. Notably, the concentration of tocopherols in the oil is exceptionally high (2965.8 mg/kg total content). These elevated levels indicate that *A. vulneraria* is a rich source of natural antioxidants and may enhance the stability of the lipid fraction during storage [1].

Sterol and tocopherol composition of the glyceride oil from the plant *A. vulneraria* is presented in Table 3. The oil demonstrates an exceptionally diverse sterol profile.

The results show that *β*-sitosterol was dominant (67.8%), followed by 24-methylenecholesterol (9.9%) and stigmasterol (8.7%). This composition places it among the sterol rich vegetable oils, particularly when compared with typical values reported for legumes. Yang et al. [22] reported that soybean oil contains approximately 60% *β*-sitosterol, 20% stigmasterol, and 10% campesterol. The content of campesterol in crude oils in Codex-Stan 210 [19] was declared to range from 6.4 to 38.6%. Tocopherols are distributed across several individual classes (*α*-tocopherol, *α*-tocotrienol, *β*-tocopherol, *γ*-tocopherol, *β*-tocotrienol, *δ*-tocopherol, *δ*-tocotrienol), with *α*-tocopherol and *α*-tocotrienol predominating at >30%. The content of *α*-tocotrienol in the analyzed sample was found to be similar to that declared in Codex-Stan 210 [19] about palm oil (25–30%).

There are no specific data on the phospholipid content of *A. vulneraria*, but it is well established that phospholipid levels in plants vary widely, and some species accumulate substantially higher amounts compared to conventional seed oils. The phospholipid profile of *A. vulneraria* indicates high biological activity, as the total phospholipid content in the oil extracted from the whole plant (6.3%) is markedly higher than the typical values reported for seed oils such as soybean and canola oil, where it was between 1.0 and 3.0% [23]. According to Thuy et al. [24], the total phospholipid content in the seed oil of the *Dalbergia tonkinensis* (Fabaceae family) was found to be 18.29%, which is about three times higher than our reported value.

**Figure 1:**
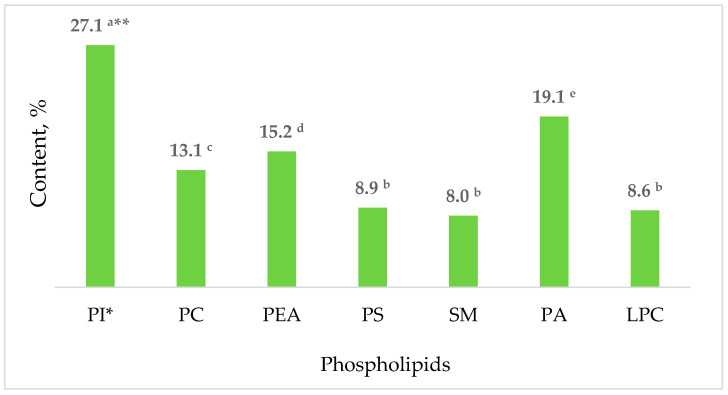
Individual composition of the phospholipid fraction of *A. vulneraria*. * PI—Phosphatidylinositol; PC—Phosphatidylcholine; PEA—Phosphatidylethanolamine; PS—Phosphatidylserine; SM—Sphingomyelin; PA—Phosphatidic acids; LPC—Lysophosphatidylcholine. ** Different letters mean significant differences between the results.

The predominant components of the individual phospholipid composition are phosphatidylinositol (27.1%) and phosphatidic acids (19.1%) (Figure 1). Phosphatidylinositol is a key membrane phospholipid involved in signal transduction, while phosphatidic acids participate in growth regulation and stress-related processes. The quality of phosphatidylethanolamine was found to be 15.2%, while the content of phosphatidylcholine was 13.1%. The presence of lysophosphatidylcholine (8.6%) and sphingomyelin (8.0%) further suggests potential applicability in dermatological formulations, particularly those targeting inflammatory conditions and tissue regeneration.

The fatty acid composition of the oil extracted from the plant *A. vulneraria* was also studied. The data are presented in Table 4, and the chromatogram is given in the Supplementary Materials.

The results indicate a high proportion of saturated fatty acids (59.4%), which represents an unusually elevated SFA content for Fabaceae family species, where unsaturated fatty acids typically predominate. This observation is not consistent with the findings of Petkova et al. [20], who reported that UFAs dominated the triacylglycerol fraction of *Vicia ervilia* (bitter vetch) seed oil, accounting for 73.4%, while SFAs comprised only 26.6%. In the studied oil, the main saturated fatty acid fraction is palmitic acid (30.3%), followed by stearic acid (8.4%). The high content of palmitic acid has a positive effect on increasing the oxidative stability of the oil and its application for non-food purposes. Monounsaturated fatty acids, which improve the lipid profile, are represented mainly by oleic acid (11.5%), and among polyunsaturated fatty acids, linoleic (5.0%) and linolenic (2.8%) are lower in content than saturated ones. According to Knothe et al. [25], the typical fatty acid profile of legume seed oils is dominated by polyunsaturated fatty acids (40–70%), mainly represented by linoleic acid (26.5–57.7%). The content of saturated fatty acids is low (10–25%), mainly represented by palmitic acid (7.7–25.3%). The iodine value was determined to be 31.7 g I_2_/100 g, which is close to cocoa butter (34.2–40.7 g I_2_/100 g), and it was similar to palm mid-fraction (34–55 g I_2_/100 g) and stearin (22–49 g I_2_/100 g) [26].

Based on the obtained fatty acid composition, an important ratio in the glyceride oil was calculated—UFA/SFA, SFA/MUFA, PUFA/SFA, and n-6/n-3. The UFA/SFA ratio determines the degree of unsaturation of the lipids; in the present case, it is 0.68. This signifies that the saturated fatty acids in the examined oil prevailed. This ratio is often used as a nutritional quality index and as an indicator of lipid functionality in foods. The SFA/MUFA ratio can be used to assess the thermal stability and health value of lipids, and for *A. vulneraria* oil, it is 2.27. The PUFA/SFA ratio in the examined samples is 0.24, which determines the high oxidative stability of the lipids, because PUFAs are more prone to oxidation. On the other hand, a higher PUFA/SFA ratio is considered beneficial for human health, especially for the reduction in incidents of cardiovascular risk [27]. The ratio of n-6/n-3 in the samples is 0.86; its lower value (closer to 1:1–4:1) is associated with anti-inflammatory and cardioprotective effects [28].

## 3. Materials and Methods

### 3.1. Materials

The aerial parts of *Anthyllis vulneraria* L. were analyzed as a combined sample. The plant was collected from Sveti Nikola (Shipka), Balkan Mountains (Stara Planina), at an altitude of 1326 m a.s.l., Bulgarka Nature Park, Stara Planina floristic region. Coordinates: 42°45′22″ N, 25°19′20″ E. Herbarium material from *A. vulneraria* was deposited under No. 063735 in the herbarium of the Agricultural University of Plovdiv (SOA).

The plant material was harvested in June 2025 during the flowering stage. The fresh parts were cleaned and air-dried under natural conditions in a shaded, well-ventilated environment at room temperature to moisture contents of 10.6%. Subsequently, the dried material was ground using a GRINDOMIX GM 200 laboratory mill (Retsch GmbH, Haan, Germany) into a 30-mesh. All aerial parts of the plant were ground together. The ground material was then subjected to analysis.

### 3.2. Chemical Composition

Lipid extraction was performed using Soxhlet extraction (a classical laboratory method) with the organic solvent *n*-hexane (boiling range 40–60 °C, CAS No. 110-54-3, Merck, Darmstadt, Germany). After extraction was completed, the solvent was removed on a rotary vacuum evaporator, and the remaining residue in the flask represented the crude glyceride oil [29].

The protein content of the plant material was quantified by the Kjeldahl method, using a Velp Scientifica digestion system (Usmate Velate, Italy) and subsequent distillation on a UDK 127 unit, in accordance with AOAC [30] guidelines. Protein content was calculated using the nitrogen conversion factor: %N × 6.25.

Total carbohydrates were calculated as follows: 100 − (weight in grams [protein + lipids + moisture + ash] in 100 g of dry plant material) [31].

The contents of fiber, ash, and moisture were determined according to AOAC [30] standard procedures. The fiber content was determined after sequential acid and alkaline hydrolysis. Approximately 3 g of plant material were treated with 1.25% H_2_SO_4_ for 30 min, filtered through a glass crucible, and washed with hot distilled water. After the acid treatment, the residue was subjected to an alkaline digestion with 1.25% KOH for 30 min. Then, it was filtered again, washed with distilled water, and dried to constant weight to obtain the crude fibers. The ash content was determined by combusting the plant material in a muffle furnace at 600 °C until a constant weight was reached. The moisture content was determined after drying the material at 105 °C until a constant weight was reached.

The energy value (EV) expressed in kcal per 100 g of sample was calculated using the conventional conversion factors: EV (kcal/100 g) = (% proteins × 4.0) + (% carbohydrates × 4.0) + (% lipids × 9.0).

### 3.3. Lipid Composition

#### 3.3.1. Fatty Acid Composition

The fatty acid profile of glyceride oil was analyzed by gas chromatography (GC) following transesterification of the oil samples in accordance with ISO 12966-1:2014 [32] and ISO 12966-2:2017 [33]. About 100 mg of the glyceride oil was methylated with methanol in the presence of H_2_SO_4_ for 2 h, after which the resulting fatty acid methyl esters (FAME) were subjected to GC analysis after purification using thin-layer chromatography (TLC). Briefly, the residue of the FAMEs was applied onto a silica gel plate and placed in a chromatographic chamber. The mobile phase consisted of petroleum ether and diethyl ether (97:3, *v*/*v*). After development, the plates were removed, air-dried, and exposed to iodine vapor. Under these conditions, the FAME bands became visible, were scraped off, and eluted with petroleum ether. The eluate was concentrated and subjected to GC analysis. The analyses were performed on an Agilent 8860 GC system (Santa Clara, CA, USA) equipped with a flame ionization detector (FID) and a DB-Fast FAME capillary column (Agilent, Santa Clara, CA, USA; 30 m × 0.25 mm × 0.25 μm). The chromatographic conditions were as follows: initial column temperature 70 °C (held for 1 min), followed by a temperature increase of 5 °C/min to 250 °C with a final hold of 3 min; injector temperature 270 °C; detector temperature 300 °C. Identification of individual fatty acids was carried out using a certified standard mixture of fatty acid methyl esters (37-component FAME Mix, Supelco, Bellefonte, PA, USA), analyzed under identical GC conditions.

#### 3.3.2. Sterol Composition

The oil samples were saponified, and the unsaponifiable fraction was isolated in accordance with ISO 18609:2000 [34]. Sterols were separated by thin-layer chromatography (TLC) and quantified spectrophotometrically following the procedure of Ivanov et al. [35]. The composition of individual sterols was analyzed using an Agilent 8860 gas chromatograph (Santa Clara, CA, USA) equipped with a DB-5 capillary column (25 m × 0.25 mm × 0.25 μm). The temperature program was as follows: initial temperature of 90 °C (held for 3 min), increased at 15 °C/min to 290 °C, followed by a further increase at 4 °C/min to 310 °C, with a final hold of 10 min. The injector and detector temperatures were set at 300 °C and 320 °C, respectively. Identification of sterols was performed using authenticated standards, including *β*-sitosterol (75%) and campesterol (10%) from Acros Organics (Morris Plains, NJ, USA), cholesterol from Acros Organics, and stigmasterol from Sigma-Aldrich (St. Louis, MO, USA), following ISO 12228-1:2014 [36].

#### 3.3.3. Tocopherols

The total tocopherol content, as well as the individual tocopherol homologues, was determined by high-performance liquid chromatography (HPLC) using a Merck-Hitachi system (Burladingen, Germany) equipped with an F-1050 fluorescence detector. Separation was achieved on a Nucleosil Si 50-5 column (250 mm × 4 mm). The mobile phase consisted of *n*-hexane and 1,4-dioxane (96:4, *v*/*v*) (*n*-hexane, CAS No. 110-54-3; 1,4-dioxane, CAS No. 123-91-1; Merck, Darmstadt, Germany), following the procedure described in ISO 9936:2016 [37].

#### 3.3.4. Phospholipids

Individual phospholipid classes were separated using two-dimensional TLC, and their concentrations were quantified spectrophotometrically, following the procedures described by Schneiter and Daum, and by ISO 10540-1:2014 [38,39].

### 3.4. Statistical Analysis

All analyses were conducted in triplicate (*n* = 3), and the results are presented as mean ± standard deviation (SD). The results for the individual sterol, tocopherol, and phospholipid composition were subjected to one-way ANOVA followed by Tukey’s test for multiple comparisons (*p* < 0.05) using IBM Statistics 19.

## 4. Conclusions

This study is significant because it represents the first investigation of the lipid fraction of *A. vulneraria* and, for the first time, the biologically active components present in the isolated oil—namely, fatty acids, sterols, tocopherols, and phospholipids—have been identified. Despite its lower yield compared to conventional seed oils, the preliminary lipid characterization of this plant, which has not yet been investigated, provides valuable insight into its fatty acid composition and highlights its potential as a source of bioactive lipids. It was established that the glyceride oil from this plant was abundant in sterols and tocopherols, in which the main components were *β*-sitosterol, *α*-tocopherol, and *α*-tocotrienol. Although the main fatty acids in the fraction were detected to be palmitic acid, the ratios of UFA/SFA (0.68) and n-6/n-3 (0.86) are below 1, which determines the lipids from *A. vulneraria* to possess potential health benefits. Other than that, the elevated content of palmitic acid also determines the oil to be oxidatively stable. Based on investigations into the chemical and lipid composition of *A. vulneraria*, it can be suggested that the plant may have potential applications in the food, cosmetic, and pharmaceutical industries. However, further studies are needed to experimentally validate these potential applications and confirm the functional significance of the identified bioactive compounds, including in vitro antioxidant and anti-inflammatory assays, cytotoxicity and biocompatibility tests, stability studies, and assessments specific to the application, such as formulation behavior in cosmetic matrices or oxidative stability in food systems.

## Figures and Tables

**Table 1:** Proximate chemical composition of *A. vulneraria*.

Compounds, %	Content, Fresh Basis	Content, Dry Weight
Glyceride oil	2.3 ± 0.3	2.7 ± 0.3
Protein	13.7 ± 0.2	15.4 ± 0.2
Total carbohydrates	67.9 ± 0.7	76.0 ± 0.8
Crude fiber	31.0 ± 0.3	34.7 ± 0.4
Ash	5.5 ± 0.1	5.9 ± 0.2
Energy Value, kcal/100 g	223.1 ± 0.4	251.1 ± 0.5

All analyses were conducted in triplicate (*n* = 3), and the results are presented as mean ± standard deviation (SD).

**Table 2:** Biologically active components from *A. vulneraria* glyceride oil.

Components	Content
Unsaponifiable matter in oil, %	28.5 ± 0.3
Unsaponifiable matter in plant, %	0.66 ± 0.10
Sterols in oil, %	7.8 ± 0.1
Sterols in plant, %	0.18 ± 0.03
Tocopherols in oil, mg/kg	2966 ± 20
Tocopherols in plant, mg/kg	68.2 ± 9.4
Phospholipids in oil, %	6.3 ± 0.5
Phospholipids in plant, %	0.15 ± 0.03

All analyses were conducted in triplicate (*n* = 3), and the results are presented as mean ± standard deviation (SD).

**Table 3:** Individual sterol and tocopherol composition of *A. vulneraria* glyceride oil.

Individual Sterols	Content, %	Individual Tocopherols	Content, %
Cholesterol	1.4 ± 0.2 ^f^*	*α*-tocopherol	31.3 ± 0.3 ^a^
Brassicasterol	0.3 ± 0.1 ^g^	*α*-tocotrienol	34.9 ± 0.5 ^b^
Campesterol	4.7 ± 0.2 ^e^	*β*-tocopherol	7.3 ± 0.2 ^d^
Stigmasterol	8.7 ± 0.3 ^c^	*γ*-tocopherol	18.7 ± 0.3 ^c^
24-Methylenecholesterol	9.9 ± 0.2 ^b^	*β*-tocotrienol	1.3 ± 0.1 ^g^
*β*-Sitosterol	67.8 ± 0.5 ^a^	*δ*-tocopherol	4.1 ± 0.2 ^e^
Sitostanol	7.0 ± 0.2 ^d^	*δ*-tocotrienol	2.4 ± 0.1 ^f^
Δ^7^-Stigmasterol	0.2 ± 0.1 ^g^		

* All analyses were conducted in triplicate (*n* = 3), and the results are presented as mean ± standard deviation (SD). Different letters in a column mean significant differences between the results.

**Table 4:** Fatty acid composition of *A. vulneraria* glyceride oil.

Fatty Acids		Content, %
Caprylic	C 8:0	1.2 ± 0.1
Undecanoic	C 11:0	4.7 ± 0.2
Myristic	C 14:0	2.7 ± 0.1
Myristoleic	C 14:1 n-5	9.3 ± 0.3
Palmitic	C 16:0	30.3 ± 0.5
Palmitoleic	C 16:1	4.1 ± 0.1
Hexadecadienoic	C 16:2 n-4	1.2 ± 0.1
Heptadecenoic	C 17:1 n-7	1.3 ± 0.1
Stearic	C 18:0	8.4 ± 0.2
Oleic	C 18:1 n-9	11.5 ± 0.3
Linoleic	C 18:2 n-6	4.2 ± 0.1
Linolenic	C 18:3 n-3	5.0 ± 0.4
Arachidic	C 20:0	2.8 ± 0.2
Behenic	C 22:0	3.8 ± 0.1
Docosadienoic	C 22:2 n-6	1.9 ± 0.1
Docosahexaenoic	C 22:6 n-3	2.1 ± 0.1
Lignoceric	C 24:0	5.5 ± 0.3
Saturated fatty acids (SFA)		59.4 ± 1.7
Unsaturated fatty acids (UFA)		40.6 ± 1.6
Monounsaturated fatty acids (MUFA)		26.2 ± 0.8
Polyunsaturated fatty acids (PUFA)		14.4 ± 0.8
Σ n-6		6.1 ± 0.2
Σ n-3		7.1 ± 0.5
Ratio between different classes of fatty acids		
UFA/SFA		0.68 ± 0.01
SFA/MUFA		2.27 ± 0.01
PUFA/SFA		0.24 ± 0.01
n-6/n-3		0.86 ± 0.03
Iodine value, g I_2_/100 g		31.7 ± 1.5

All analyses were conducted in triplicate (*n* = 3), and the results are presented as mean ± standard deviation (SD).

## Data Availability

The original contributions presented in this study are included in the article and Supplementary Materials. Further inquiries can be directed to the corresponding author.

## Supplementary Materials

The following supporting information can be downloaded at: https://www.mdpi.com/article/10.3390/plants15131954/s1, S1: Chromatogram of fatty acid methyl esters of glyceride oil from kidney vetch (*Anthyllis vulneraria* L.).
