# Supplementary material for: Preliminary Study on Nutritional Value and Biologically Active Components of Kidney Vetch (Anthyllis vulneraria L.)
Source: Plants (Basel). 2026 Jun 25;15(13):1954. doi: 10.3390/plants15131954 (PMC13364288; doi:10.3390/plants15131954)

Supplementary Material S1. Chromatogram of fatty acid methyl esters of glyceride oil from kidney vetch (*Anthyllis vulneraria* L.)

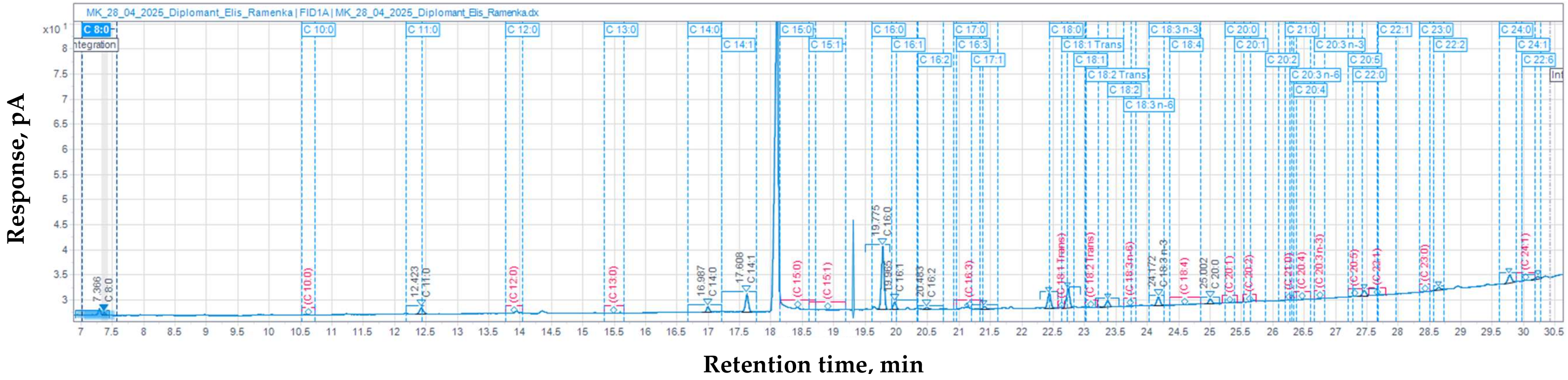

Supplement: Supplementary file 1 [file plants-15-01954-s001.zip › plants-4345684-supplementary.pdf]
